# Enabling Transformational Leadership to Foster Disaster-Resilient Hospitals

**DOI:** 10.3390/ijerph20032022

**Published:** 2023-01-22

**Authors:** Heba Mohtady Ali, Jamie Ranse, Anne Roiko, Cheryl Desha

**Affiliations:** 1 Cities Research Institute, Griffith University, Gold Coast and Brisbane, QLD 4215, Australia; 2 School of Engineering and Built Environment, Griffith University, Brisbane, QLD 4215, Australia; 3 Department of Emergency Medicine, Griffith University, Gold Coast, QLD 4215, Australia; 4 Menzies Health Institute, Griffith University, Gold Coast, QLD 4215, Australia

**Keywords:** hospital leaders, transformational leadership, adaptation, recovery, resilience, regeneration, climate change, disasters, model, self-reflection assessment checklist

## Abstract

Hospitals’ operational performance during disasters varies from failing, to being responsive and resilient, to dealing with disruption and surprise. Transformational leaders enable continuously learning hospitals that are resilient in the face of disasters by adapting regeneratively and evolving beyond undertaking conventional lesson-learning after each disaster. However, learning from successful transformational leaders in healthcare is still ad hoc with a lack of guidance on how to develop such leaders. Hence, this study sought to identify key competencies of transformational leaders by exploring hospital leaders’ actions in dealing with disasters, considering the disaster cycle of prevention, preparedness, response, and recovery (PPRR). A qualitative case-study design was adopted comprising in-depth semi-structured interviews with twelve senior hospital staff with operational leadership experience with disasters. Three significant categories (themes) and seven key component competencies (sub-themes, in brackets) of transformational leaders were revealed through the analysis of transcripts: (1) ‘Governance and leadership’ (‘transformative agency’ and ‘decisive accountability’); (2) ‘Planning and risk assessment’ (‘risk navigation’, ‘disaster attunement’, and ‘planning agility’); and (3) ‘Communication and network engagement’ (‘communication accelerator’ and ‘collaboration innovator’). The authors propose a transformational leadership model for hospital disaster resilience and an assessment checklist for leaders’ self-reflection to support hospitals in their transition to resilient operations.

## 1. Introduction

Hospitals are required to be operational and provide essential services for patients in an ongoing arrangement, including during disasters of all types [1,2]. This includes natural disasters such as floods, earthquakes, and hurricanes, as well as human-made disasters such as chemical spills, terrorism, power outages, and conflicts. Climate change is making a range of natural disasters more likely and more severe, alongside the challenges of the COVID-19 pandemic [3,4]. Indeed, ‘disasters’ is a term often used to encapsulate the incidents brought about by factors such as climate change, geopolitics, tectonics (such as earthquakes and volcanic eruptions) and biological disruptions (such as epidemics and pandemics) [5]. A reliable indicator of a hospital’s ‘resilience’ to disasters is its ability to keep the system serviceable and ‘business as usual’ with negligible disruptions while responding to, and recovering from, one or more concurrent disasters [4,6,7]. Following disasters, a ‘resilient hospital’ could conceivably recover beyond its original level of performance, ‘transforming’ in the process to a new way of delivering its essential services [8,9].

Resilience engineering provides a useful lens through which to consider hospitals’ ability to deal with disasters by anticipating, responding to, monitoring, and learning from these situations. This includes considering capacity for adaptation—making changes to the existing system, and transformation—making more significant changes or creating a new system [10,11,12]. The ‘PPRR’ (prevent–prepare–respond–recover) model is also a useful framework that has been used globally and prolifically in Australia to explore disaster resilience, through the key phases of ‘Preparedness’, ‘Prevention’, ‘Response’, and ‘Recovery’, in addition to ‘anticipation’ and ‘assessment’ [8,11,13,14].

A variety of capacities (i.e., ability to deliver) are readily identified in the literature to explain hospitals that are ‘resilient’ in facing disruption and uncertainty. This includes, for example, being ‘preventive’ (mitigation and preparedness), ‘anticipative’ (planning for the future), ‘absorptive’ (resistant, prepared, and able to withstand disruption), ‘adaptive’ (using alternate reserves or processes while providing services), and ‘restorative’ (recovering rapidly and at sensible cost). Other resilient hospital capacities have been identified as ‘transformative’ (restorative with better readiness and reduced vulnerability for future risk) [8,10,11,15,16,17]. Additionally, hospitals can be ‘learning’ (reflecting and re-viewing past success and failure) to apply gained knowledge and experiences, inform future strategies and policies, and change existing learning approaches and organisational culture [15,16]. Hospitals that transform into regenerative learning organisations can survive and utilise emerging and hidden opportunities [18,19,20]. Hence, regeneration includes revitalizing human resources along the way. This can be achieved through the provision of comprehensible, manageable, and meaningful work, which promotes personal and professional development and helps individuals sustainably cope with future challenges [21,22].

Hospital resilience requires hospital leaders to have particular ‘competencies’ (i.e., the ability to do something successfully) in bringing about the organizational capacities noted above. In times of disaster, hospitals may struggle to meet the increased demand for care due to a shortage of staff and resources. Hospital leaders must find ways to overcome these operational challenges to ensure that patients receive the care they need, including novel approaches to plan, respond to, and manage disasters [3,4,23]. Clinical professionals may lack the essential evidence-based knowledge about specific successful leadership styles, effectiveness in disaster management, and the competencies to apply their gained knowledge and skills [24,25,26]. ‘Transformational leadership’ is a recommended approach for healthcare leaders to make their hospitals more resilient and has been identified as an effective style for clinical professionals [20,26,27]. It is centered around the relationship between leaders and their subordinates, with the goal of improving their work experiences.

Transformational leaders utilize techniques such as idealized influence, motivation, intellectual stimulation, and individualized consideration to achieve superior results. This style of leadership relies on the emotional intelligence of the leader [24,26,27,28]. Numerous competencies have been previously documented to guide hospital leaders in disaster planning and management [26,27,29,30]. For example, the American Organization of Nurse Leaders (AONL) established competencies for nurse executives and leaders in three vital areas: communication, business, and leadership [30]. Additionally, twelve key emergency leadership capabilities emerged from the key informant in a study conducted by Caro [1]. Leadership has been one of the main components of the Hybrid Resilience Learning Framework (HRLF), which was previously created by the authors of this paper for evaluating both hospital’s organisational resilience and learning from disasters [15].

In spite of the widely understood benefits of having transformational leaders within the health sector, the emergence of such leadership is ad hoc rather than intentional. Additionally, there is a lack of guidance on how to develop such competencies, with transformational leadership competencies still poorly identified and relatively unexplored [29,31,32,33,34]. Hence, this study sought to identify key competencies of transformational leadership in hospitals, exploring hospital leaders’ actions in dealing with disasters, considering the disaster ‘PPRR’ cycle of prevention, preparedness, response and recovery. The authors asked, “How can a hospital leader be transformational to enhance the hospital’s resilience to disasters?”. Building on previous research [15,35], the authors were particularly interested in the role of ‘learning’ by hospital leaders and how the construct of resilience could be a productive framing for hospitals to augment ‘recovery’ efforts post-disaster.

## 2. Methods

### 2.1. Design

This study adopted a qualitative case-study approach, including in-depth and semi-structured interviews. Interviews were designed to enable participants to reflect on their leadership experiences while anticipating, responding to, monitoring, and learning from climate change impacts and disasters [36,37]. Their perceptions were sought about their reflections on factors that contributed to their success and challenges that hindered their success while responding to disasters, and any other insights gained through experiences in dealing with disasters as a senior hospital staff member.

### 2.2. Participant Recruitment

One hospital is in the Gold Coast and the other in Brisbane; both are very densely populated parts of Southeast Queensland, Australia. These hospitals were selected to ensure that both were exposed to similar challenges, had comparable opportunities, hierarchies, and political regime management structures, and followed the same policies. Both hospitals provide public healthcare services, health education and research, surgery, trauma, general and specialist medicine, maternity and emergency medicine, intensive care, outpatient care, and other services. Each of these hospitals serves more than 50,000 patients per year and has more than 8000 staff. To ensure confidentiality, throughout this article, we have used (H1) and (H2) to report on these two hospitals. 

The research team liaised with two hospital advisors (one for each hospital) to discuss the recruitment of and to ensure the questions and logistics for the study were appropriate for the hospital workers. The hospital advisor in one hospital was the disaster and emergency management coordinator, and in the other hospital, the advisor was a nurse manager (nursing executive). The responsibility of each of the advisors was the chief point of contact for the research team’s activities with their hospitals.

The two advisors distributed information about the study and the research team’s contact information to potential senior hospital staff interviewee participants via email. The sampling methods were limited to the services and departments approved for the project by liaising with the hospitals’ management. All these senior hospital staff should have had leadership experiences through disasters and climate change impacts. The researchers engaged with interested staff about logistics via email, phone calls, and a meeting invitation. Interviews occurred either face-to-face or virtually (using Microsoft TEAMS) based on the interviewee’s preferences. 

### 2.3. Data Collection and Analysis

Interviews were conducted confidentially by two researchers (the first author was the leader, and one of the co-authors was an observer) between June 2021 and January 2022. Each interview lasted approximately 45–60 min.

With the participant’s permission, each interview was audio recorded using the Microsoft Teams recording function and one of the researchers’ mobile phones was used to record a backup copy. Each interview recording was transcribed verbatim by the first author using Microsoft Word and saved in a file format compatible with NVivo. Participants’ details, interview transcripts, and recordings were coded with a participant number for discussion and publication purposes and kept in confidence and accessible only by the researchers listed on the ethics approval list.

Thematic analysis was used to analyse the data from the interview transcripts using Braun and Clarke’s framework as a guide [38]. This was supported by the features within the NVivo software, enabling a detailed and nuanced account of the data to be produced. Themes and subthemes considered shared components of previously identified competencies, capabilities, and frameworks in literature, including governance and leadership, planning and risk assessment, emergency preparedness, communication, collaboration, decisiveness, transformational skills, learning, and evaluation [1,15,30].

The coding themes for this study were drawn from the HRLF (Hospital Resilience Leadership Framework) learning areas, and from the leadership competencies outlined in Caro’s 2016 study, with subthemes drawn from both the HRLF and Caro’s leadership competencies [1,15]. In addition, actions related to resilience were taken into account, including those aimed at reducing the impact of a disaster and returning to a stable state after the event. These actions were based on the disaster cycle framework (PPRR) and the principles of resilience engineering, which focus on anticipating, responding to, and learning from disasters [8,11]. We focused on five key resilience capacities: anticipative, preventive, absorptive, adaptive, and transformative, which are seen as essential for resilience, and may be supported by skills such as learning, regenerating, organizing, and resourcefulness [10].

### 2.4. Quality Assurance

The validity or trustworthiness of the data analysis was addressed by ensuring transparency and consistency in the coding process [39]. An audit trail was provided of how and when any codes were created or renamed and how key themes were identified through coherence and exemplified appropriately. The research team discussed the meanings and interpretations of the codes and themes to minimise biased reporting and identify areas where information was likely to be missing.

Recruitment for data collection was stopped when it was determined that no new themes were being identified. The identified themes were organized into content domains and a matrix was used to identify patterns and connections among the domains. The final analysis was also reviewed by two individuals who had not been involved in the initial coding process. 

### 2.5. Model and Checklist Development

Drawing on the literature review findings and analysis of the interview results, a model was created to contextualize the key competencies of transformational leadership to enable the actions required before, during, and after disasters, for resilient hospitals. The model considered the resilient capacities of hospitals, which were identified through the literature and used in the analysis of results [8,11,17]. A checklist was also created to support leaders to undertake a self-assessment of their actions, enabling individual learning and professional development. The checklist items were informed by the Disaster Cycle Framework and the Principles of Resilience Engineering [14,40], which focus on anticipating, responding to, and learning from disasters. 

Both the model and the checklist considered actions related to reducing the impact of a disaster and returning hospital operations to a stable state after the event. This includes addressing key aspects of resilience, leadership, disaster management, and the ability of a hospital to withstand, recover, and adapt to disruptions.

## 3. Results

Out of 15 invited senior hospital staff members, 12 were available to be interviewed. All the participants were over 18 years of age, including 6 females (3 from each hospital) and 6 males (3 from each hospital). The participants included hospital directors, managers, and consultants. The disasters that many participants mentioned were floods (6 participants), COVID-19 (5 participants), aged care closure (4 participants), and power outages (2 participants). Other disasters that were mentioned once include bushfires, cyclones, and problems with water or sterilisation. Most participants discussed their experiences in the given hospitals, and a few connected their reflections with other related experiences elsewhere.

Analysis of the results included three significant competencies (themes) present in transformational leadership: (1) ‘Governance and leadership’; (2) ‘Planning and risk assessment’; and (3) ‘Communication and network engagement’. These themes draw on the authors’ previous paper [15]. Within the three themes, seven sub-themes were used to distil participants’ views on the roles and competencies required of hospital leaders to achieve a resilient hospital. The themes and subthemes are illustrated in Table 1 below and discussed in the following sections.

### 3.1. Theme 1: Governance and Leadership

Table 2 shows the competencies (sub-themes) and participant exemplars linked to governance and leadership. It includes two competencies: transformative agency and decisive accountability.

### 3.2. Theme 2: Risk Assessment and Planning

Table 3 shows the competencies (sub-themes) and participant exemplars linked to risk assessment and planning. It includes three competencies: risk navigation, disaster attunement, and planning agility.

### 3.3. Theme 3: Communication and Engagement

Table 4 shows the competencies (sub-themes), and participant exemplars linked to leaders’ communication and engagement. It includes two competencies: communication accelerator and collaboration innovator.

## 4. Discussion

The themes for this research analysis were derived from the HRLF, and sub-themes were drawn from both the HRLF and Caro’s leadership competencies [1,15]. Additionally, the study considered actions related to resilience, including those that aim to reduce the impact of a disaster and return to stability after the event. These actions were informed by the PPRR disaster cycle framework and principles of resilience engineering, which emphasize anticipating, responding to, and learning from disasters [11,14,40].

This analysis led to significant findings about the governance and leadership of hospitals, including the importance and challenges of change and transformation, the difficulties of making decisions in uncertain and complex situations, and the role of governance structure, hierarchy, and ecosystem (as shown in Table 2). The risk assessment indicated that anticipating worst-case scenarios, such as vulnerabilities and potential losses, is crucial in disaster planning. Disaster planning should consider various scenarios and situations, have contingency plans in place, and prioritize prevention and preparedness. Leaders can benefit from learning from others’ experiences as well as their own past experiences (as shown in Table 3). Effective communication, being well-prepared, and keeping regular communication with team members were identified as key success factors for hospital leaders during disasters. Communication challenges included confusing or misdirected information, overwhelming tasks, and debriefing that did not involve everyone. The strategic collaboration included sharing and discussing information and seeking assistance from experts and skilled team members (as shown in Table 4).

Several participants in our study identified the impact of transformational skills and changing mindset (H1.S4, H1.S1, H2.S2, H1.S6, and H2.S6) (see Table 2). Speedily changing situations call for a leader who can be a major change agent. [41]. Moreover, “Systems actualizing calls upon regenerative practitioners to work towards fulfilling their potential as change agents to enable communities to optimise the development of interrelated systems. To be regenerative, the emphasis must be on building their own capability and systems stewardship” [42] (p. 948).

Many participants described the significance of their effective communication and strategic collaborations with their teams as strategies towards successful leadership and achievements (H1.S5, H1.S6, H2.S4, H2.S5, and H2.S6) (see Table 4). Transformational leaders are examples of relationally focused leadership styles, as they apply idealized influence, inspiration and motivation, intellectual stimulation, and individualized consideration to achieve superior results, communicate vision and mission, and use intellectual stimulation to support and challenge employees [24,26,27]. Under such transformational leadership, physicians positively perform tasks, solve problems, and surpass difficulties in their daily routine function and consequently enhance their job performance [34].

The effectiveness of transformational leaders stems from their ability to create collaboration, communicate shared values, and organize collective action [43]. Participants highlighted the magnitude of accountability and decisiveness (H1.S1, H1.S4, H2.S2, H2.S3, H2.S4, and H1.S6), risk assessment, planning, and preparedness in managing disasters (H1.S2, H1.S3, H1.S4, H2.S4, and H2. S5) (see Table 2, Table 3 and Table 4). Leaders can influence people to accomplish assigned tasks and achieve goals cooperatively, professionally, and effectively [44].

Half of the participants described how planning and making decisions can be driven by self-learning based on prior knowledge and gained experiences or by learning from others’ experiences (H1.S2., H1.S3, H1.S4, H1.S5, H2.S4, and H2.S5). Leaders should lead by example and adopt continuous learning as a critical strategy for transformation because it establishes platforms and opportunities for deep shared learning and can evolve thinking and leadership while transitioning towards an extremely diverse future [45].

Disasters drain the routine operational healthcare delivery and capacity [46]. Leadership becomes an adaptable developmental process where different situations necessitate adopting various approaches by the leaders [44,47]. Several leadership styles were identified in the literature without a consensus regarding which styles are the most effective [26,47]. The positive impact of transformational leadership has been identified within the healthcare sector [29,31,32,33]. Transformational leadership positively and significantly impacts adaptive culture and organisational resilience and, therefore, it was identified as one of the successful styles for clinical professionals [20,26,27]. However, there is scarce information regarding the recommended competencies for such leaders in managing hospitals during disasters to ensure the resilience, learning, and regeneration of these hospitals. Within this context, the current study explored these key competencies and roles.

### 4.1. The Value of Transformational Leadership

Disasters and climate change impacts have been proven to adversely affect healthcare workers’ physical and mental health [35]. Studies have demonstrated the significance of visionary leadership within the healthcare system in urban contexts, particularly regarding the coordination and alignment of efforts among various stakeholders. This encompasses not only leaders within the healthcare system but also those in the public, private, and community sectors. Strong leadership can aid in the resolution of complex public health issues in urban areas through the establishment of effective relationships with leaders beyond the healthcare system [48,49,50]. Healthcare and hospital leaders must reconstruct health systems and monitor their performance towards sustainable recovery and ‘Build-Back-Better’ [51]. These leaders should not only make their organisations well-prepared to manage extreme, recurrent, and detrimental climate change emergencies but also consider a more sustainable and greener approach to reduce greenhouse gas emissions and carbon production [4]. Transformational leaders are recommended to lead hospitals as they motivate others to perform higher than their original intentions and exceed their limits and self-thoughts. Moreover, better staff retention, organizational commitment, job satisfaction, physical health, and mental wellness were reported with transformational leadership [26,27,52].

### 4.2. Transformative Leadership for Hospital Disaster Resilience

Learning from past failures and successes is a significant component of a resilient organisation; it is the ‘Factual’ component of the resilience engineering approach. Nevertheless, within hospitals and healthcare systems, there is a gap between organisational learning and institutionalised practice [15,40]. Leaders can influence people to accomplish assigned tasks and achieve goals cooperatively, professionally, and effectively [44]. Thinking beyond recovery towards a disaster-resilient hospitals requires transformational leaders who can lead continuously learning, adapting, and evolving organisations [4,51].

**Figure 1:**
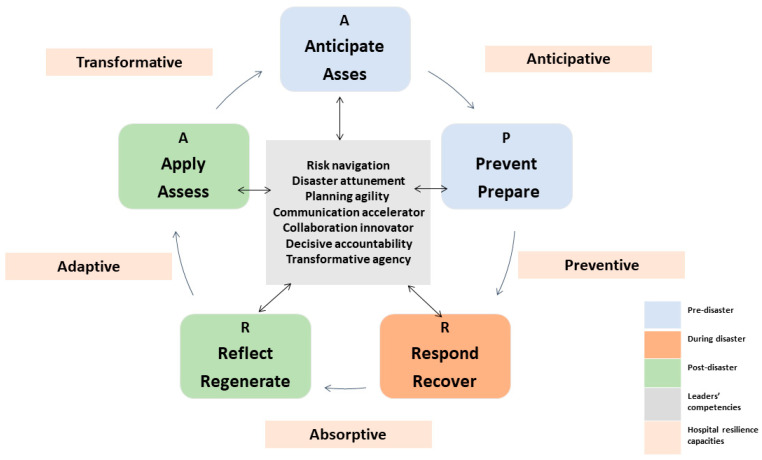
Transformational leadership model for hospital disaster resilience-‘APRRA model’.

Given that health services contribute to climate change and are responsible for up to 5% of the world’s greenhouse gas emissions [53,54], this study proposes a ‘transformational leadership model for hospital disaster resilience‘ called ‘APRRA’ as a shift towards a disaster regenerative transformational leadership. This model is grounded by the hospital resilience capacities and recommended transformational leaders’ competencies. Additionally, it shows the actions that should be taken accordingly (A (anticipate and assess), P (prevent and prepare), R (respond and recover), R (reflect and regenerate) and A (apply and assess again)). The implementation of the ‘APRRA model’ can facilitate a transformative shift in the operational paradigm of hospitals, wherein leaders are imbued with the requisite competencies to effectively manage disaster-related situations. As a result, leaders are able to make informed and appropriate decisions during the pre-disaster, disaster, and post-disaster phases, thereby augmenting the establishment of crucial resilience capacities within the hospital environment. Additionally, the ‘APRRA’ model enables leaders to utilize their gained knowledge and experiences in re-assessing resilience and risk assessment to lead the hospital towards a transformative resilience capacity beyond the ‘PPRR’ recovery approach (See Figure 1).

### 4.3. A Transformational Hospital Leaders Assessment (THLA) Checklist: Toward Disaster Resilient Hospitals

Self-aware, genuine leaders usually have high emotional intelligence and are motivated by their intrinsic self-actualizing requirement [55]. Personified self-awareness is a fundamental feature for people to steer the disturbance and uncertainty ahead and a higher level of consciousness is required to transcend their biases and self-interest in order to act for shared collective values and goals [45,56]. The transformational leader’s ability to exercise self-awareness is an essential factor contributing to their success and effectiveness, as it establishes collaboration, communicates shared values, and mobilises collective action [43].

The authors of this study have also developed the Transformational Hospital Leaders Assessment (THLA) Checklist, presented in Table 5. This checklist is a practical self-assessment tool to evaluate transformation learning from disasters. It emerged supported by the components of the APRRA model and the significance of self-awareness as a contributor to the success of transformational leaders. The ‘THLA checklist’ serves as a valuable tool for leaders to continually evaluate and improve their performance in real time and through reflection, contributing to self-improvement and sustained resilience in hospital disaster management. The developed ‘THLA checklist’ should be further assessed and validated in subsequent research studies.

The literature on resilience has established that it encompasses both the process and outcome aspects. This implies that both process and outcome indicators are essential for measuring resilience [2,10]. The authors propose a few indicators to support measuring the effectiveness and efficiency of the approaches and actions proposed in the ‘APRRA model’ model and ‘THLA checklist’ such as: the number of disasters anticipated timely and properly; the number of actions that improved hospital disaster preparedness; the number of effective disaster responses and satisfactory recovery; the number of debriefing sessions following disasters; the number of disaster plans containing lessons learned from previous disasters; and the number of long-lasting changes/policy modifications based on gained prior knowledge [10].

## 5. Limitation of the Study

Given the topic area of disaster resilience, there was a risk that the researchers may impact political or institutional sensitivities regarding classified information, public awareness campaigns, and past events. To minimise this potential, the researchers ensured that political and institutional sensitivities formed part of the discussions with the project advisors prior to each round of interviews. Moreover, there were the limitations of in-depth interviews being not generalisable, time-consuming, requiring a more prolonged verification process, and difficult to add context. It is challenging to explore the extent to which individuals have personally been affected by disasters or have actively participated in climate change response efforts during these interviews. Finally, it would be beneficial to include a comparative aspect in future research to increase the thoroughness and gain further understanding. This will be taken into consideration when planning for future studies.

## 6. Conclusions

In a rapidly changing world where disasters are becoming more frequent and in-tense, hospitals are faced with a timely choice in protecting and enabling their workforce and assets to serve communities herein. Hospital leaders have an unprecedented responsibility to manage their organizational planning, adaptation, and recovery from the diversity and severity of disasters that their hospitals experience and respond to. The findings of this research have immediate application in the health sector for hospital organizational resilience. The ‘APRRA model’ enables a transformed hospital operational environment where leaders are equipped with disaster-related management competencies. Applying the model, leaders can affect appropriate actions before, during, and after disasters, enabling the delivery of essential hospital resilience capacities at the appropriate time. The ‘THLA checklist’ provides an important due diligence continual learning mechanism for leaders to evaluate their own performance, in real-time and on reflection, towards self-improvement and sustained hospital disaster resilience outcomes.

## Figures and Tables

**Table 1:** Transformational leadership categories (themes) and competencies (subthemes).

Key Category (Theme)	Category Scope	Key Competency (Sub-Theme)	Competency Description
Governance and leadership	Policies, management, administration, regulation, decision making, and accountability.	1.1. Transformative agency	Enabling space for changing ideas, thoughts, and movement in organizational culture.
		1.2. Decisive accountability	Taking on accountability, decision making, and managing fast-paced situations.
2. Planning and risk assessment	Approaches and strategies undertaken, lessons applied, and challenges addressed.	2.1. Risk navigation	Anticipating different scenarios and situations and assessment of possible vulnerabilities, threats, and hazards.
		2.2. Disaster attunement	Adapting approaches in planning and preparedness based on risk assessment and various situations and considerations.
		2.3. Planning agility	Self-directed modifications in planning and making decisions, based on prior knowledge and gained experiences.
3. Communication and network engagement	Significance, challenges, and approaches towards communication effectiveness with co-workers, colleagues, and the community.	3.1. Communication accelerator	Facilitating vertically integrated and inter-professional communication across the organization.
		3.2. Collaboration innovator	Rewarding inter-professional engagement and collaboration with co-workers within and between organizational units.

**Table 2:** Governance and leadership.

Summarized Description	Participants’ Excerpts	The Actions That Should Be Taken in the Disaster Cycle Frame	Hospital Resilience Capacities
1. Transformative agency:			
Five participants described the significance and challenges of change management and how disaster should be managed from a leadership perspective by using tools such as change management training as follows:			
The significance of change and transformation	*“The importance of putting in a disaster framework and that we needed to change the way that we ran decision-making compared to normal business” (H1.S4).* *“You had to be a lot stricter on your decision-making and communication with staff… we needed to change the way that we ran decision-making compared to normal business” (H1.S4).*	Reflect and regenerate	Adaptive
The challenges of change management	*“Training people to be flexible, which is hard because some people find that really difficult. Especially when they are stressed, and sleep deprived and scared” (H1.S1)* *“The traditional line of a chain of command and the traditional decision-making process is very slow and cumbersome” (H1.S4).* *“It really does come down to having the right people with the right experience around the table to be able to get make a decision” (H2.S2).* *“You’re always alert for changes … for you need to change your response depending on [what] comes down the line, and then you go back out and re-communicate in the new way” (H1.S6).* *“To balance all these patients in one place, I found that quite challenging” (H2.S6).*	Respond and recover	Absorptive
1.2. Decisive accountability:			
Five participants highlighted the challenges in the decision-making process, including governance structure, hierarchy, and ecosystem. Additionally, these challenges included complex and evolving situations and uncertainty as follows:			
Governance structure, hierarchy ecosystem	*“The usual governance structures and chains of command aren’t always effective at dealing with these things, which is why you need to have an operations controller” (H1.S4).* *“Whilst …the broader health ecosystem didn’t step in, there were some players who tried to step in, who didn’t understand the hierarchy, that made it challenging that we had to sort of, actually say, back off” (H2.S2).*	Respond and recover	Absorptive
Complex and evolving situations	*“We think of complex adaptive systems, but essentially particularly in the setting of a disaster, you have a very complex situation with many moving parts not all working in the same duration in the early phases of a disaster and how you can you know obviously commanding control is one thing” (H2.S4).* *“We had a goalpost health” (H2.S2).* *“We had to make some decisions on what to do with those patients quite quickly, in what was a very rapidly evolving situation” (H2.S6).*	Reflect and regenerate	Adaptive

**Table 3:** Risk assessment and planning.

Summarized Description	Participants’ Excerpts	The Actions That Should Be Taken in the Disaster Cycle Frame	Hospital Resilience Capacities
2. Risk navigation (what if):			
Four participants explained how they anticipated different scenarios and situations and assessed possible vulnerability, threats, and hazards as follows:			
Loss of a unit/a function	*“If we were to lose our intensive care unit, for instance, we would move all those folk, … We can set the endoscopy suite up as a redundant ICU. We can report redundant capacity, so hopefully, we would not have to … evacuate our ICU to another hospital because that places people at terrible risk” (H1.S3).* *“We knew a lot about what we could possibly expect” (H1.S2).*	Anticipate and assess	Anticipative
Loss of staff	*How would we manage with way less staff? And what services we would turn off and pull back on in order to be able to maintain critical inpatient services?” (H1.S4).*		
Being vulnerable	*“Power is power and oxygen and things like that are obviously vulnerabilities for hospitals…you need more than one power source … so it has got five generators …but they all go through the one switch port, so you just need one live rat that becomes a dead rat, and that whole switchboard goes down…” (H1.S2).*		
2.2. Disaster attunement:			
Four participants discussed approaches adopted in planning based on risk assessment and various situations and considerations as follows:			
Having different scenarios and situations	*“How we got all the people treated well, the same time, plan how we fixed the problem. So, there were sort of two streams of planning. One was looking after the patients and the second was planning how we were going to fix the problem” (H1.S3).* *“We all have business continuity plans that are well established. And that really takes you through case scenarios that you might face, and what you need.” (H2.S4).* *“Hospital has its own disaster management plan and then we have a whole lot of specific Disaster responses … And it’s all quite well mapped out for us to help us anticipate the risks” (H1.S2).* *“I think where you can buy yourself time by doing the key things that you’re likely to do is good; essentially, another word for that would be contingency planning” (H2.S4).* *“It’s very hard to write a manual or procedure or process to be able to cover everything and possible scenario that come in” (H2.S5).*	Anticipate, assess, prevent and prepare	Anticipative Preventive
Focusing on prevention and preparedness	*“The* *prevention* *, preparation, response, recovery and so the first two are the most important ones for reducing the negative impacts and enhancing. a positive impact” (H1.S2).*		
2.3. Planning agility:			
Five participants discussed how planning and making decisions can be driven by self-learning based on prior knowledge and gained experiences or by learning from others’ experiences, e.g., other members of the team or other organisations or even countries, as follows:			
Case scenarios Benchmarking Past experiences	*2.3.1.* *learning from others’ experiences*	Prevent, prepare, apply and assess	Preventive Transformative
	*“Some of the information we were getting was not helpful in that it was sort of scaring the life out of us, and we were sort of looking at all these worst-case type scenarios that did not end up happening, but I think obviously if that hadn’t played out, it would have been really useful to have that information. I think we were preparing for the worst” (H1.S4).* *“* *Benchmarking* *becomes critical in those sorts of times. Finding out what other facilities are doing and how they are managing things and what works and what doesn’t work, I think, was critical” (H1.S4).* *We were able to stand on the experience of everybody else in Europe and the UK and so on… if this were a bomb or a Gas leak or something, you might not haven’t got this sort of luxury” (H1.S2).* *“If it’s not one individual or if there’s a core group of people that have some past experience, as long as they’re heard vowed to utilize their past experiences, navigates a pathway to provide a clear outcome, as opposed to use the example of business continuity plans, we’ll have this document is prepared, but if it’s prepared by someone who is not a clinician or doesn’t have experience to try and implement something that doesn’t fit or mold or if it’s so rigid that it can’t be adapted”(H2.S5).*		
Similar past experiences (cookie cutter) Previous complex situations	*2.3.2.* *learning from self-experience*	Reflect and regenerate	Adaptive
	*“We had some confidence that we’d be able to manage this as a project and get the place back on track, so* *that* *was really interesting exercise.…it became a nice cookie cutter for the management of these sorts of things” (H1.S2).* *“I had managed quite a number of complex situations before and so drawing on that experience was vital in responding to this situation. That is probably a key” (H1.S3).* *“I think it just* *comes* *from experience again. I look at hospitals or a disaster every day; if you are not prepared, things will fall over. …And I suppose it is just experienced, and it is looking at how we can get the best out of the staff that we have got at the time and using their skills to the best of their ability to be able to support the services and to build that resilience into the workforce” (H1.S5).* *“In the moment when you deploy to sites, you know, think about you having aged care facility or always COVID. I tend to draw on my experience in managing disasters and that’s been my professional background for 20 years” (H2.S4).*		

**Table 4:** Communication and engagement.

Summarized Description	Participants’ Excerpts	The Actions That Should Be Taken in the Disaster Cycle Framework	Hospital Resilience Capacities
3.1. Communication accelerator:			
3.1.1. *Communication significance:*			
Two participants discussed how leaders can best communicate, the significance of effective, timely communication, and the challenges that hinder it as follows:			
Significance of being well-prepared and good communicators	*“Having the communication is the key” (H1.S5).* *“There is a bit of a formula that I follow as a calm professional about what are the things that I need to prepare in the event of a disaster, …what are the three things that we’re going to be saying about this publicly, but other than that, no list of criteria that I use to answer the question” (H2.S4).*	Prevent and prepare	Preventive
Keeping regular communication with the teams	*“The main thing is communication and communication with the team of what is going on. If you get them on board and you communicate with them regularly … you will have them behind you” (H1.S5).*	Respond and recover	Absorptive
3.1.2. *Communication challenges*:			
Three participants discussed the challenges that hinder the effectiveness of communication during disasters as follows:			
Confusing and improperly directed information	*“Where things got very confused in this disaster was that people weren’t communicating up the right avenues and down the right avenues. And we had a lot of information coming in on the sides and people, doing things that were not directed to…it’s where people lose sight in and disaster to forget to communicate to the teams well” (H1.S5).* *“If the command does not flow through quickly enough from the department to support that decision-making or those in action, or it does not end up being enacted, then you have done something that upset people. And thinking you are doing the right thing. But then you know you are not backed up” (H1.S6).*	Respond and recover	Absorptive
Overwhelming tasks	*“And that is a very big community interface and a lot of management of people you know at the front door, and so you’ve got your people standing at the front of the hospital saying you can’t come in and having to deal with irate people” (H1.S6).*		
Debriefing did not include everyone	*“There was a debrief that happened at an executive level, but my team that was actually managing the scenario weren’t invited to it, and we don’t know the outcomes from their debrief, to see how we need to change our practice…so, we don’t know if we did well if there was an opportunity to learn what corrective actions are being put in place” (H2.S5)*	Reflect and regenerate	Adaptive
3.2. Collaboration innovator:			
Three participants discussed how leaders collaborate with their co-workers and team members as follows:			
Sharing and discussing the information	*“I am always conscious of if I hear something, making sure that I share that information with the risk team, and then now work with those with that expert or with that particular area to work that risk up and to say, …, this was mentioned in a board meeting as a potential problem that the chief executive was worried about. Tell us about it. What are you doing to mitigate those risks? What is the cause of the risk”? (H2.S4).* *“We kind of learning on the go to start with…you could have to think completely differently about it, but we had a lot of wise heads in the room and a lot of experienced people” (H1.S6).* *“We learned together what to do, and we learn together what to do, and we learn from each other. So other facilities learned from listening to the way other people were doing it, and we learned from the external agencies” (H1.S6).*	Prevent, prepare, respond, and recover	Preventive and absorptive
Getting help from experts and skilled team members	*“You will not have every skill required in that scenario. But knowing your team’s skill set and knowing how to utilize that” (H2.S6).*	Apply and assess	Transformative
	*“It was a good bonding moment for me and my nurses, is, like, Alright, can you guys can help me? I don’t know how to do this” (H2.S6).).*	Reflect and regenerate	Adaptive

**Table 5:** Transformational Hospital Leaders Assessment (THLA) Checklist: Toward Disaster Resilient Hospitals.

Phase of Disaster			Leadership Assessment Questions	Evaluation * (Y)(T)(N)	Comments **
**Pre-disasters**	A	Anticipate	Did you anticipate this disaster?		
		Assess	Did you have any form/documented risk assessment actions?		
	P	Prevent	Were you able to prevent any of the predictable hazards?		
		Prepare	Were you and your team prepared?		
			Did you have a specific disaster plan?		
			Was your team involved in developing this plan as a bottom-up approach?		
			Did you share this plan with your team?		
**During disaster**	R	Respond	Was the disaster response successful?		
		Recover	Did your organization return to their business as usual (BAU)?		
**Post-disaster**	R	Reflect	Did you have the opportunity to debrief and reflect on what happened during this disaster?		
			Did you share the experience gained with your team, department, or organisation?		
		Regenerate	Did you consider sustainability and green approaches while making decisions and/or recovering?		
			Will you include sustainable development goals in your upcoming plans?		
	A	Apply	Did you apply what you learnt from this experience in your disaster planning?		
		Assess	Did you use what you learnt from this disaster (gained knowledge and skills) to assess your hospital’s resilience?		
			Did you use what you learnt from this disaster (gained knowledge and skills) to assess any anticipated risk?		

* (Y): yes, (T): to some extent, (N): no. ** (e.g., How/Why did you do it? How/Why did you not do it?).

## Data Availability

The data supporting the results are in the form of interview transcripts and can be made available by written request to the corresponding author.
